# Polymorphism of the Dopa-Decarboxylase Gene Modifies the Motor Response to Levodopa in Chinese Patients With Parkinson's Disease

**DOI:** 10.3389/fneur.2020.520934

**Published:** 2020-10-29

**Authors:** Lanting Li, Huixia Lin, Ping Hua, Lei Yan, Hui Dong, Tan Li, Weiguo Liu

**Affiliations:** ^1^Department of Neurology, The Affiliated Brain Hospital of Nanjing Medical University, Nanjing, China; ^2^Department of Medical Genetics and Cell Biology, School of Basic Medical Sciences, Zhengzhou University, Zhengzhou, China; ^3^Department of Neuro-Psychiatric Institute, The Affiliated Brain Hospital of Nanjing Medical University, Nanjing, China

**Keywords:** Parkinson's disease, polymorphisms, *DDC*, levodopa, motor response

## Abstract

Levodopa (L-DOPA) is the most effective drug for Parkinson's disease (PD). However, the response to L-DOPA remains individually variable, which hampers the practical value of L-DOPA in the clinic. Genetic factors play a role in L-DOPA efficacy. This study explored the associations between polymorphisms and motor response to L-DOPA in Chinese patients with PD. A total of 51 Chinese PD patients were enrolled in this study. Patients underwent an acute L-DOPA challenge and were evaluated by the Unified Parkinson Disease Rating Scale (UPDRS) part III at baseline and after L-DOPA administration. Subjects were genotyped for polymorphisms: rs921451 and rs3837091 in the *DDC* loci, rs3836790 in the *SLC6A3* locus, rs4680 in the *COMT* locus, and rs1799836 in the *MAOB* locus. We found that patients carrying the *DDC* CT or TT genotype exhibited a better motor response to L-DOPA than patients with the *DDC* CC genotype, and there was still a significant difference after adjustment for the L-DOPA dose in the acute challenge. Improvement in the UPDRS III subscores, including bradykinesia and axial symptoms, was significantly lower in patients with the *DDC* CC genotype than in patients with the CT or TT genotype. There were no significant associations between the motor response to L-DOPA and the rs3837091, rs3836790, rs4680, and rs1799836 variants. The *DDC* single nucleotide polymorphism rs921451 modulated the motor response to L-DOPA in Chinese PD patients. Our results suggested that *DDC* may be a modifier gene for the L-DOPA treatment response in PD.

## Introduction

Parkinson's disease (PD) is a common neurodegenerative disorder affecting more than 1% of the population over 60 years old (1). The main pathological hallmark is the loss of dopaminergic neurons in the substantia nigra pars compacta (2). PD is characterized by bradykinesia, rigidity, tremor and other non-motor symptoms, including depression, cognitive disorders, sleep disorders (3). Current treatment is based on different dopamine replacement strategies and is aimed at symptomatic relief. Levodopa (L-DOPA) has been the gold standard in the treatment of PD since its production in 1960 (4). However, the response to L-DOPA remains individually variable, which hampers the practical value of L-DOPA in the clinic.

Dopaminergic pathway genes have been investigated, and some single nucleotide polymorphisms (SNPs) have been reported to be associated with L-DOPA efficacy. The *DDC* gene encodes a key enzyme, aromatic L-amino acid decarboxylase (AAAD), involved in the synthesis of dopamine in the central nervous system (CNS) and the periphery. AAAD plays an important role in the generation of an effective dose of L-DOPA, which may cause interindividual variations in treatment response. The polymorphisms in the *DDC* gene have been considered to affect the expression of AAAD and thus induce clinical heterogeneity (5, 6). In addition, rs921451 and rs3837091 in the *DDC* gene promoter have been frequently investigated and concluded to influence diseases involved in the mesolimbic dopamine system, such as smoking (7–9) and bipolar affective disorder (6, 10, 11). Additionally, ethnic differences were demonstrated in these studies. The associations between polymorphisms in the *DDC* gene and PD have also been explored (12). However, few studies have focused on the association between polymorphisms in the *DDC* gene and the response to L-DOPA. The influence of these polymorphisms on the response to L-DOPA is subject to debate.

Polymorphisms in other dopaminergic pathway genes, including *SLC6A3, COMT* and *MAOB*, have also been considered to be important factors in the response to L-DOPA. Most dopamine reuptake occurs through solute carrier family 6, member 3 (SLC6A3, also called DAT1) back to presynaptic neurons, which is the most powerful determinant of dopamine neurotransmission (13). *SLC6A3* SNP rs3836790 has been reported to be associated with the L-DOPA treatment response (14). Catechol-*O*-methyltransferase (COMT, encoded by the *COMT* gene) and monoamine oxidase B (MAOB, encoded by the *MAOB* gene) are the main enzymes involved in dopamine metabolism. The most commonly studied polymorphisms are rs4680 in the *COMT* gene and rs1799836 in the *MAOB* gene, which regulate the enzyme activity (15, 16). The correlation between these genotypes and motor response to L-DOPA revealed paradoxical results.

Here, we conducted a study of Chinese PD patients to explore the association between polymorphisms in the dopaminergic pathway genes and the response to L-DOPA by acute challenge experiments. To the best of our knowledge, there have not been published reports of pharmacogenetic modulation of the motor response to an acute L-DOPA challenge in Chinese patients.

## Materials and Methods

### Subjects

Fifty-one PD patients were recruited from the Movement Disorder Clinic at the Department of Neurology of the affiliated Brain Hospital of Nanjing Medical University from July 2015 to October 2018. All PD patients were examined by at least two movement disorder specialists according to the United Kingdom Parkinson's Disease Society Brain Bank criteria (17). The exclusion criteria included atypical PD, dementia, treatment with clozapine or monoamine oxidase inhibitors, serious or unstable medical disorders. The study was approved by the ethics committee of the affiliated Brain Hospital of Nanjing Medical University. All participants signed an informed consent.

### Procedures

We performed an acute L-DOPA challenge in the fasting state after 24 h of withdrawal of dopaminergic agonists, 12 h of withdrawal of L-DOPA and other antiparkinsonian medications. The L-DOPA dose administered for the challenge corresponded to 150% of the usual morning L-DOPA equivalent dose used by patients (i.e., first morning dose of L-DOPA, plus the L-DOPA equivalent dose of the first morning dose of dopamine agonist, plus an additional 50 mg of L-DOPA). The L-DOPA used in our study was immediate-release L-DOPA with 25% benserazide. Patients were allowed to have a light breakfast 20–30 min after dosing.

Patients were evaluated by the Unified Parkinson Disease Rating Scale (UPDRS) (18) part III at baseline and after L-DOPA administration. UPDRS III was divided into subscores for tremor (UPDRS items 20 and 21), rigidity (UPDRS item 22), bradykinesia (UPDRS items 23–26 and 31) and axial symptoms (UPDRS items 27–30) (19). The primary efficacy criterion included the improvement in the UPDRS part III motor score (ΔUPDRS III), the improvement in subscores (Δtremor, Δrigidity, Δbradykinesia, Δaxial symptoms) after acute L-DOPA administration, relative to baseline. The basic clinical features were also recorded, including age, sex, age at onset, duration of the disease, Hoehn and Yahr scale score (H-Y), L-DOPA equivalent dose (LED), Mini-Mental State Examination (MMSE), and Montreal Cognitive Assessment (MOCA). The LEDs of patients were calculated according to a previous study (20).

### Genotyping

Genomic DNA was extracted from venous blood samples by the use of a TIANamp Blood DNA Kit (TIANGEN, China) according to the manufacturer's instructions. Subjects were genotyped for polymorphisms: rs921451 and rs3837091 in the *DDC* loci, rs3836790 in the *SLC6A3* locus, rs4680 in the *COMT* locus, and rs1799836 in the *MAOB* locus. Primers for polymerase chain reaction (PCR) amplification were designed using Primer3 (http://primer3.ut.ee/), and the sequences of the primers are shown in Supplementary Table 1. The cycling conditions for amplification were as follows: 2 min at 94°C, 35 cycles of 15 s at 94°C, 15 s at 60°C and 1 min at 72°C and then an extra 10 min at 72°C. The amplification products of five polymorphisms were sequenced on an ABI 3730xl automated sequencer (Applied Biosystems, Foster City, CA, USA). Reference sequences were obtained from the PubMed website.

### Statistical Analysis

Continuous variables were expressed as the median and 25th−75th percentile range. Hardy-Weinberg equilibrium was tested using the chi-squared test. Dominant, recessive, and additive genetic models were used for analysis depending on the genotype frequencies. The dominant genetic model was the comparison between the group of subjects carrying at least one minor allele and the group of non-minor allele carriers, while the recessive genetic model was the comparison between the group of homozygous carriers of minor allele and the group of subjects carrying the other genotypes. Additive genetic model was the comparison among the groups of subjects carrying different genotypes. Comparisons between groups were performed using the nonparametric Mann-Whitney *U* test or Kruskal-Wallis test. Adjustments for the L-DOPA dose were made using nonparametric analysis of covariance. Because of the location of *MAOB* on the X chromosome, men and women were analyzed separately. All analyses were 2-tailed, and the level of statistical significance was set at *p* < 0.05.

## Results

### Clinical Characteristics and Genotype Distributions of all PD Patients

A total of 51 subjects were included in the study. The mean age and age at onset of the PD patients were 61.7 and 55.2 years (SD = 7.8 and 8.4, respectively), with an average of 6.7 years duration of disease and 35% female. The mean UPDRS part III motor score (off L-DOPA) and H-Y score were 40.1 and 2.6 (SD = 16.2 and 0.9, respectively).

The distributions of all variants complied with Hardy-Weinberg equilibrium (Table 1). For rs921451, 12 patients had the TT genotype, 27 had the CT genotype, and 12 had the CC genotype. Patients with the CC genotype (*n* = 12) and patients with the CT or TT genotype (*n* = 39) did not differ significantly in terms of baseline characteristics (Table 2). There were no intergroup differences in the frequencies of *COMT* genotypes.

**Table 1:** SNP genotype distributions.

**SNP**	**Gene**	**Major/Minor allele**	**MAF^**b**^**	**Genotype**	**Frequencies, *n* (%)**	**HWE, *p*-value**
rs921451	*DDC*	T/C	0.47	CC	12 (23.5)	0.916
				CT	27 (53.0)	
				TT	12 (23.5)	
rs3837091	*DDC*	AGAG/–	0.51	–/–	16 (31.4)	0.636
				AGAG/–	28 (54.9)	
				AGAG/AGAG	7 (13.7)	
rs3836790	*SLC6A3*	NA	NA	4R/4R	5 (9.8)	0.052
				4R/5R	11 (21.6)	
				5R/5R	35 (68.6)	
rs4680	*COMT*	G/A	0.28	AA	4 (7.8)	0.880
				AG	18 (35.3)	
				GG	29 (56.9)	
rs1799836^a^	*MAOB*	A/G	0.18	GG	3 (16.7)	0.801
				GA	7 (38.9)	
				AA	8 (44.4)	
				G	6 (18.2)	NA
				A	27 (81.8)	

*SNP, single nucleotide polymorphism; MAF, minor allele frequency; HWE, Hardy–Weinberg equilibrium; NA, not available*.

a *Men and women were calculated separately*.

b *Based on the allele frequency of East Asian from NCBI SNP database*.

**Table 2:** Baseline characteristics of the patients in each group.

**Genotype**	**rs921451**		
	**CC (*N* = 12)**	**CT + TT (*N* = 39)**	***p*-value**
Age (years)	62.5 (58.3–66.8)	63.0 (57.0–67.0)	0.956
Gender ratio (F/M)	3/9	15/24	0.502
Age at onset (years)	57.5 (54.0–63.3)	56.0 (49.0–60.0)	0.301
Disease duration (years)	3.5 (2.0–6.0)	7.0 (4.0–10.0)	0.063
H-Y (off L-DOPA)	2.0 (2.0–2.5)	3.0 (2.0–3.0)	0.202
Baseline UPDRS part III motor score (off L-DOPA)	29.0 (22.5–45.5)	40.0 (29.0–50.0)	0.063
Usual L-DOPA daily dose (mg/day)	300.0 (200.0–375.0)	300.0 (300.0–600.0)	0.185
Usual total L-DOPA daily dose equivalent (mg/day)	300.0 (256.2–462.5)	400.0 (300.0–625.0)	0.205
L-DOPA dose in the acute challenge (mg)	200.0 (200.0–200.0)	200.0 (200.0–300.0)	0.324
MMSE	28.0 (24.3–29.8)	28.0 (27.0–29.0)	0.743
MOCA	24.5 (20.5–26.0)	25.0 (21.0–26.0)	0.947

*Values were expressed as the median and 25th-75th percentile range except for the gender category. UPDRS, Unified Parkinson Disease Rating Scale; H-Y, Hoehn and Yahr scale score; MMSE, Mini-Mental State Examination; MOCA, Montreal Cognitive Assessment*.

### Association Between Genotypes and the Motor Response to L-DOPA

In a recessive model, the ΔUPDRS III was significantly lower in patients carrying the *DDC* CC genotype than the CT or TT genotype (*p* = 0.025) (Table 3). When adjusted for the L-DOPA dose in the acute challenge, there was still a significant difference (*p* = 0.048). The Δbradykinesia and Δaxial symptoms were significantly lower in patients with the *DDC* CC genotype than the CT or TT genotype (*p* = 0.037 vs. 0.003). After adjusting for the L-DOPA dose, no significant differences were found in terms of UPDRS III subscores (*p* = 0.096 vs. 0.076). In addition, there were no significant associations between the response to L-DOPA and the rs3837091, rs3836790, rs4680 and rs1799836 variants (Table 3).

**Table 3:** Effect of genotypes on the improvement of UPDRS motor score following administration of L-DOPA in recessive models.

**SNP**	**Gene**	**Genotype**	**ΔUPDRS III**	**ΔTremor**	**ΔRigidity**	**ΔBradykinesia**	**ΔAxial symptoms**
rs921451	*DDC*	CC	8.5 (8.0–15.0)	2.5 (0.3–5.3)	3.0 (2.0–4.0)	4.0 (2.3–5.8)	0.0 (0.0–1.0)
		CT + TT	16.0 (11.0–27.0)	3.0 (1.0–7.0)	5.0 (1.0–6.0)	7.0 (4.0–10.0)	2.0 (1.0–5.0)
		*p-*value	0.025^*^	0.420	0.224	0.037^*^	0.003^**^
		*p*^b^*-*value	0.048^*^	0.114	0.263	0.096	0.076
rs3837091	*DDC*	–/–	10.5 (8.0–25.3)	2.0 (0.0–4.5)	3.0 (1.3–5.8)	4.0 (3.0–11.5)	1.5 (0.3–3.0)
		AGAG/– +AGAG/AGAG	14.0 (11.0–25.0)	3.0 (1.0–7.0)	4.0 (2.0–6.0)	6.0 (4.0–9.0)	1.0 (0.0–4.0)
		*p*-value	0.238	0.161	0.540	0.476	0.925
		*p*^b^-value	0.334	0.192	0.435	0.584	0.719
rs3836790	*SLC6A3*	4R/4R	18.0 (9.5–26.0)	0.0 (−0.5–7.0)	6.0 (2.0–7.5)	7.0 (3.5–8.5)	3.0 (1.0–5.0)
		4R/5R + 5R/5R	13.0 (8.8–25.5)	3.0 (1.0–7.0)	3.5 (1.8–6.0)	6.0 (4.0–10.0)	1.0 (0.0–3.0)
		*p*-value	0.703	0.292	0.279	0.899	0.243
		*p*^b^-value	0.878	0.959	0.462	0.659	0.715
rs4680	*COMT*	AA	10.5 (4.5–15.8)	2.0 (0.3–6.0)	2.5 (−0.8–5.8)	4.0 (3.3–4.8)	1.0 (0.3–1.8)
		AG + GG	14.0 (9.0–27.0)	3.0 (1.0–7.0)	4.0 (2.0–6.0)	6.0 (4.0–10.0)	1.0 (0.0–4.0)
		*p*-value	0.194	0.646	0.350	0.140	0.399
		*p*^b^-value	0.191	0.821	0.264	0.147	0.224
rs1799836^a^	*MAOB*	GG	13.0 (NA)	2.0 (NA)	7.0 (NA)	4.0 (NA)	1.0 (NA)
		GA + AA	18.0 (12.0–32.0)	3.0 (1.0–7.0)	5.0 (1.0–7.0)	9.0 (4.0–12.0)	3.0 (1.0–7.0)
		*p*-value	0.906	0.857	0.439	0.552	0.549
		*p*^b^-value	0.482	0.628	0.878	0.371	0.323
		G	16.0 (11.0–21.8)	1.0 (−0.3–4.5)	5.0 (2.6–6.0)	6.5 (5.8–9.5)	1.0 (0.0–3.5)
		A	12.0 (8.0–21.0)	3.0 (1.0–7.0)	3.0 (1.0–5.0)	5.0 (3.0–8.0)	1.0 (1.0–2.0)
		*p*-value	0.512	0.231	0.145	0.145	0.791
		*p*^b^-value	0.608	0.610	0.138	0.213	0.909

*Values were expressed as the median and 25th-75th percentile range. NA, not available due to small numbers of patients with MAOB GG genotype*.

a *Men and women were calculated separately*.

b *Adjusted for the L-DOPA dose in the acute challenge*.

* *p < 0.05*.

** *p < 0.01*.

In a dominant model, improvement in rigidity was higher in patients with the *MAOB* GG or GA genotype than AA genotype among women (*p* = 0.010). After adjustment for the L-DOPA dose in the acute challenge, this association was still significant (*p* = 0.022). No significant associations were found between the response to L-DOPA and the rs921451, rs3837091, rs3836790, and rs4680 variants in dominant models (Supplementary Table 2). In the additive models, genotypes for rs921451 and genotypes for rs1799836 in females were associated with the improvement in UPDRS III subscores (*p* = 0.012 vs. 0.033). However, there were no significant differences when adjusted for the L-DOPA dose (*p* = 0.203 vs. 0.061). Therefore, there were no relationships between the response to L-DOPA and polymorphisms in additive models (Supplementary Table 3).

## Discussion

This study first presented the association of polymorphisms with acute response to L-DOPA effects in Chinese PD patients. The clinical response to L-DOPA is generally evaluated after chronic administration with the drug. However, this response is subject to many sources of factors. It has been suggested that acute L-DOPA challenge could be used to predict long-term drug efficacy (21, 22). Thus, acute L-DOPA challenge is a better marker of the response to dopaminergic medication than chronic administration. Our results showed that patients who carry the CT or TT genotype in the *DDC* have a better response to L-DOPA in motor function compared with those with the CC genotype. However, analysis of other polymorphisms (rs3837091 in the *DDC* gene, rs3836790 in the *SLC6A3* gene, rs4680 in the *COMT* gene, and rs1799836 in the *MAOB* gene) failed to show any association.

The homozygous CC genotype for rs921451 had lower effect on motor response to an acute L-DOPA challenge in Chinese PD patients. Two studies investigated the effect of rs921451 on the acute response to L-DOPA in European populations. In accordance with our study, Devos et al. (23) conducted a research on 33 PD patients and reported that rs921451 C carriers exhibited a lower response to L-DOPA without affecting its pharmacokinetics. Instead, Moreau et al. (14) found that rs921451 was not associated with the response to L-DOPA. In the latter study, the recessive model was not discussed. However, our analysis of rs921451 in a recessive model showed a significant association. Another reason for the difference may be that patients involved in the study by Moreau et al. had deep brain stimulators, severe gait disorders and longer disease duration. The homozygous CC genotype may affect the response to L-DOPA by changing the bioavailability of dopamine in the CNS (23). A decreased expression of AAAD in the CNS reduced the decarboxylation of L-DOPA into dopamine and led to lower of a motor response. The analysis of rs3837091 failed to show any association with acute response to L-DOPA in both our study and a previous study (14). A significant association was observed in another research which demonstrated that patients carrying the AGAG/AGAG genotype had better response to L-DOPA in motor function (23). The conflicting results indicated that further studies are required.

There was no relationship between the response to an acute L-DOPA challenge and *COMT* SNP rs4680 in our study. However, the results of previous studies are controversial. In a cohort of Chinese populations, the *COMT* AA genotype was associated with high daily LEDs (24). In contrast, no associations were observed between rs4680 and the L-DOPA treatment response, but there was a tendency for patients carrying the *COMT* GG genotype to need high daily LEDs (25–27). The patients with the *COMT* AA genotype recruited for the former study had a higher H-Y stage, longer disease duration, and higher UPDRS scores, which may have an influence on the association between the variant and the response to L-DOPA. Furthermore, the frequency of the *COMT* AA genotype was low, only eight patients (6%) with the *COMT* AA genotype were included. Therefore, the results require further evaluation. Bialecka et al. (28) investigated the effect of *COMT* haplotypes (formed by four *COMT* SNPs: rs6269, rs4633, rs4818, rs4680) that have been shown to influence COMT enzymatic activity, on the response to L-DOPA and identified an association between the high-activity haplotype and high daily LEDs. Cheshire et al. (29) found that patients carrying the combined *COMT* GG genotype and *MAOA* TT genotype received significantly high daily LEDs. We speculated that *COMT* SNPs may have an effect on the L-DOPA treatment response. The low COMT activity allows slower catabolism of L-DOPA and more stable serum and CNS drug concentrations, resulting in a better clinical response (28). However, the effect of a single *COMT* SNP was too subtle to be observed in a clinical setting. It seems that *COMT* haplotypes may exert a greater effect on the response to L-DOPA.

No association was found between the response to an acute L-DOPA challenge and *SLC6A3* SNP rs3836790 in our study. One study reported that rs3836790 in *SLC6A3* was associated with an acute response to L-DOPA effects. Patients with *SLC6A3* 6R/6R genotype had a better response to L-DOPA than other genotypes (14). However, the *SLC6A3* 6R allele was not found in our study, which may have caused this different outcome. In accordance with previous studies, no association was found between the *MAOB* SNP rs1799836 and the L-DOPA treatment response. The *MAOB* GG genotype seemed to be more frequent in patients with high daily LEDs (25, 27).

In conclusion, rs921451 in the *DDC* gene had an effect on the L-DOPA treatment response in Chinese PD patients. However, the effect of polymorphisms on the L-DOPA treatment response may vary due to ethnic differences. In addition, sample size is another cause of the different results. A large cohort is needed to verify the results. Our results indicated that *DDC* may be a modifier gene for the L-DOPA treatment response in Chinese PD patients and may act as a predictive marker for therapy optimization. This study provides evidence for physicians to adjust the L-DOPA dose in clinical practice using pharmacogenetic information.

## Data Availability Statement

All datasets generated for this study are included in the article/Supplementary Material.

## Ethics Statement

The studies involving human participants were reviewed and approved by the Ethics Committee of the Affiliated Brain Hospital of Nanjing Medical University. The patients/participants provided their written informed consent to participate in this study.

## Author Contributions

WL designed this study. WL, LL, PH, and LY were responsible for diagnosis. LL, PH, and LY were responsible for clinical evaluation. HD and TL performed genetic analysis. LL and HL collected and analyzed the data. LL wrote the manuscript. WL and HL contributed to the revision of the manuscript. All authors contributed to the article and approved the submitted version.

## Conflict of Interest

The authors declare that the research was conducted in the absence of any commercial or financial relationships that could be construed as a potential conflict of interest.
