# Supplementary material for: Polymorphism of the Dopa-Decarboxylase Gene Modifies the Motor Response to Levodopa in Chinese Patients With Parkinson's Disease
Source: Front Neurol. 2020 Oct 29;11:520934. doi: 10.3389/fneur.2020.520934 (PMC7673431; doi:10.3389/fneur.2020.520934)
Supplement: Supplementary file 1 [file Table_1.DOCX]

Supplementary Material

**Supplementary Table 1.** Primers for the variants in the *DDC*, *SLC6A3, COMT* and *MAOB* gene.

| SNP | Forward primer (5′→3′) | Reverse primer (5′→3′) |
| --- | --- | --- |
| rs921451  rs3837091  rs3836790  rs4680  rs1799836 | TGGCTTGTGTGGTTTGGAGT  GGAGCCTGGTCGTTCTCAAA  ACTGTGGGTTCACAGCACAT  GACCAGCGTGAGCATAGAGG  AAGACCTTTTGGCGCCTCTTA | CTTGGTTCAGTTGTGCAGCC  AATGCTCAGCTGTCCCTGTC  TTACCTGGGTCCTTTTGGGC  CACTGAGGGGCCTGGTGATA  CATGTGGAGAATCACCAGGCT |

SNP, single nucleotide polymorphism.

**Supplementary Table 2.** Effect of genotypes on the improvement of UPDRS motor score following administration of L-DOPA in dominant models.

| SNP | Gene | Genotype | △UPDRS Ⅲ | △tremor | △rigidity | △bradykinesia | △axial symptoms |
| --- | --- | --- | --- | --- | --- | --- | --- |
| rs921451  rs3837091  rs3836790  rs4680  rs1799836^a^ | *DDC*  *DDC*  *SLC6A3*  *COMT*  *MAOB* | CC+CT  TT  *p* value  *p*^b^ value  -/-+AGAG/-  AGAG/AGAG  *p* value  *p*^b^ value  4R/4R+4R/5R  5R/5R  *p* value  *p*^b^ value  AA+AG  GG  *p* value  *p*^b^ value  GG+GA  AA  *p* value  *p*^b^ value | 13.0(8.0-21.0)  16.5(12.5-30.0)  0.350  0.666  15.0(9.0-26.5)  13.0(8.0-21.0)  0.528  0.442  10.5(6.5-13.0)  16.0(8.0-32.0)  0.618  0.256  13.0(10.8-26.8)  14.0(8.0-24.5)  0.627  0.983  24.5(12.0-34.3)  12.5(7.5-27.5)  0.213  0.486 | 2.0(1.0-7.0)  4.0(0.5-7.0)  0.630  0.924  2.5(0.3-7.0)  3.0(2.0-3.0)  0.772  0.660  2.0(0.3-5.3)  3.0(1.0-7.0)  0.177  0.240  2.5(0.8-6.3)  3.0(1.0-7.0)  0.788  0.488  4.5(1.8-7.0)  2.0(0.0-3.0)  0.127  0.207 | 4.0(2.0-6.0)  4.5(0.3-6.8)  0.938  0.861  4.0(2.0-6.0)  2.0(0.0-5.0)  0.215  0.241  4.0(1.3-6.0)  4.0(2.0-6.0)  0.911  0.662  4.5(1.0-6.0)  3.0(2.0-5.5)  0.833  0.889  7.0(5.0-9.0)  3.0(0.0-4.8)  0.010*  0.022* | 5.0(4.0-8.0)  8.5(4.0-11.5)  0.270  0.444  6.0(4.0-9.8)  7.0(2.0-12.0)  0.731  0.806  7.0(3.3-8.8)  5.0(4.0-10.0)  0.879  0.458  7.0(4.0-10.0)  5.0(3.0-9.5)  0.272  0.820  7.5(4.0-12.8)  8.5(2.0-13.8)  0.858  0.815 | 1.0(0.0-3.0)  2.5(1.0-3.8)  0.265  0.731  1.0(0.0-4.8)  1.0(1.0-2.0)  0.528  0.292  1.0(0.3-3.0)  2.0(0.0-5.0)  0.406  0.195  1.0(0.0-4.3)  1.0(0.5-3.0)  0.815  0.622  4.0(0.8-7.0)  1.5(0.3-6.8)  0.686  0.876 |

Values were expressed as the median and 25th to 75th percentile range. ^a^ Genotypes in female were shown. ^b^ Adjusted for the L-DOPA dose in the acute challenge. * *p* < 0.05.

**Supplementary Table 3.** Effect of genotypes on the improvement of UPDRS motor score following administration of L-DOPA in additive models.

| SNP | Gene | Genotype | △UPDRS Ⅲ | △tremor | △rigidity | △bradykinesia | △axial symptoms |
| --- | --- | --- | --- | --- | --- | --- | --- |
| rs921451  rs3837091  rs3836790  rs4680  rs1799836^a^ | *DDC*  *DDC*  *SLC6A3*  *COMT*  *MAOB* | CC  CT  TT  *p* value  *p*^b^ value  -/-  AGAG/-  AGAG/AGAG  *p* value  *p*^b^ value  4R/4R  4R/5R  5R/5R  *p* value  *p*^b^ value  AA  AG  GG  *p* value  *p*^b^ value  GG  GA  AA  *p* value  *p*^b^ value | 8.5(8.0-15.0)  14.0(10.0-27.0)  16.5(12.5-30.0)  0.078  0.141  10.5(8.0-25.3)  16.0(12.0-26.5)  13.0(8.0-21.0)  0.307  0.355  18.0(9.5-26.0)  12.0(10.0-14.0)  16.0(8.0-32.0)  0.684  0.399  10.5(4.5-15.8)  15.5(17.8-32.3)  14.0(8.0-24.5)  0.267  0.389  13.0(NA)  27.0(18.0-33.0)  12.5(7.5-27.5)  0.367  0.471 | 2.5(0.3-5.3)  2.0(1.0-7.0)  4.0(0.5-7.0)  0.701  0.233  2.0(0.0-4.5)  3.5(1.0-7.0)  3.0(2.0-3.0)  0.373  0.306  0.0(-0.5-7.0)  2.0(1.0-3.0)  3.0(1.0-7.0)  0.363  0.436  2.0(0.3-6.0)  2.5(0.8-6.3)  3.0(1.0-7.0)  0.893  0.710  2.0(NA)  5.0(2.0-7.0)  2.0(0.0-3.0)  0.215  0.274 | 3.0(2.0-4.0)  5.0(2.0-6.0)  4.5(0.3-6.8)  0.454  0.526  3.0(1.3-5.8)  5.0(2.0-6.0)  2.0(0.0-5.0)  0.286  0.262  6.0(2.0-7.5)  3.0(1.0-5.0)  4.0(2.0-6.0)  0.425  0.526  2.5(-0.8-5.8)  4.5(1.8-6.0)  3.0(2.0-5.5)  0.554  0.528  7.0(NA)  7.0(6.0-9.0)  3.0(0.0-4.8)  0.033*  0.061 | 4.0(2.3-5.8)  6.0(4.0-9.0)  8.5(4.0-11.5)  0.101  0.247  4.0(3.0-11.5)  6.0(4.3-8.8)  7.0(2.0-12.0)  0.664  0.794  7.0(3.5-8.5)  7.0(3.0-9.0)  5.0(4.0-10.0)  0.987  0.756  4.0(3.3-4.8)  7.5(5.0-10.5)  5.0(3.0-9.5)  0.080  0.276  4.0(NA)  10.0(4.0-12.0)  8.5(2.0-13.8)  0.755  0.678 | 0.0(0.0-1.0)  2.0(1.0-5.0)  2.5(1.0-3.8)  0.012*  0.203  1.5(0.3-3.0)  1.0(0.0-5.0)  1.0(1.0-2.0)  0.817  0.467  3.0(1.0-5.0)  1.0(0.0-1.0)  2.0(0.0-5.0)  0.140  0.243  1.0(0.3-1.8)  1.0(0.0-5.0)  1.0(0.5-3.0)  0.700  0.493  1.0(NA)  5.0(1.0-7.0)  1.5(0.3-6.8)  0.653  0.612 |

Values were expressed as the median and 25th to 75th percentile range. NA, not available due to small numbers of patients with *MAOB* GG genotype. ^a^ Genotypes in female were shown. ^b^ Adjusted for the L-DOPA dose in the acute challenge. * *p* < 0.05.
